# Bioactivity and Physico-Chemical Properties of Dental Composites Functionalized with Nano- vs. Micro-Sized Bioactive Glass

**DOI:** 10.3390/jcm9030772

**Published:** 2020-03-12

**Authors:** Reto Odermatt, Matej Par, Dirk Mohn, Daniel B. Wiedemeier, Thomas Attin, Tobias T. Tauböck

**Affiliations:** 1 Department of Conservative and Preventive Dentistry, Center for Dental Medicine, University of Zurich, 8032 Zurich, Switzerland; 2 Department of Endodontics and Restorative Dentistry, School of Dental Medicine, University of Zagreb, 10000 Zagreb, Croatia; 3 Institute for Chemical and Bioengineering, Department of Chemistry and Applied Biosciences, ETH Zurich, 8093 Zurich, Switzerland; 4 Statistical Services, Center for Dental Medicine, University of Zurich, 8032 Zurich, Switzerland

**Keywords:** dental resin composites, bioactive glass filler, nanoparticles, microhardness, degree of conversion, hydroxyapatite

## Abstract

Bioactive resin composites can contribute to the prevention of secondary caries, which is one of the main reasons for failure of contemporary dental restorations. This study investigated the effect of particle size of bioactive glass 45S5 on chemical and physical composite properties. Four experimental composites were prepared by admixing the following fillers into a commercial flowable composite: (1) 15 wt% of micro-sized bioactive glass, (2) 15 wt% of nano-sized bioactive glass, (3) a combination of micro- (7.5 wt%) and nano-sized (7.5 wt%) bioactive glass, and (4) 15 wt% of micro-sized inert barium glass. Hydroxyapatite precipitation and pH rise in phosphate-buffered saline were evaluated during 28 days. Degree of conversion and Knoop microhardness were measured 24 h after specimen preparation and after 28 days of phosphate-buffered saline immersion. Data were analyzed using non-parametric statistics (Kruskal–Wallis and Wilcoxon tests) at an overall level of significance of 5%. Downsizing the bioactive glass particles from micro- to nano-size considerably improved their capability to increase pH. The effect of nano-sized bioactive glass on degree of conversion and Knoop microhardness was similar to that of micro-sized bioactive glass. Composites containing nano-sized bioactive glass formed a more uniform hydroxyapatite layer after phosphate-buffered saline immersion than composites containing exclusively micro-sized particles. Partial replacement of nano- by micro-sized bioactive glass in the hybrid composite did not impair its reactivity, degree of conversion (*p* > 0.05), and Knoop microhardness (*p* > 0.05). It is concluded that downsizing bioactive glass particles to nano-size improves the alkalizing potential of experimental composites with no negative effects on their fundamental properties.

## 1. Introduction

Although the remarkable development of dental resin composites during the last two decades had led to their mechanical properties and esthetics plateauing at a high level [1,2], the issue of secondary caries has still not been appropriately addressed [3]. Therefore, the true potential for further improvements of this material class lies in introducing the capability of preventing secondary caries formation [4]. Different approaches for introducing caries-preventive properties for restorative materials have been investigated, including the incorporation of ion-releasing fillers [5], and the addition of various antibacterial agents, such as silver [6], chlorhexidine [7], antibiotics [8], and quaternary ammonium compounds [9]. The re-occurrence of caries at restoration margins can be inhibited through specific interactions of dental materials with the oral environment, which are known under the common term “bioactivity”. Despite the lack of consensus regarding the exact definition of bioactivity of dental resin composites, one can reasonably adopt the broad understanding of the term, which includes the material’s capability to release remineralizing ions, elevate the pH, and precipitate hydroxyapatite [10]. Recent studies have shown that all of these effects can be attained by adding reactive bioactive glasses (BGs) into methacrylate-based resin composites.

Resin composites functionalized with BG are capable of releasing calcium, phosphate, and fluoride ions [11,12], which can remineralize enamel [13] as well as demineralized dentin [14]. Furthermore, BG-containing resin composites can precipitate hydroxyapatite on their surface [15], thereby sealing marginal gaps and reducing bacterial penetration [16]. The pH rise that accompanies BG dissolution exerts an antimicrobial effect [17]. Although resin composites modified with BG mostly pertain to the experimental domain, a dual-curing restorative composite containing calcium fluorosilicate BG has recently become commercially available for clinical use [18]. Besides methacrylate-based composites, other material types have also been experimentally functionalized with BG, e.g., epoxy-based root canal sealers [19], calcium silicate cements [20], and root canal dressings [21]. Micro-sized [22,23] and nano-sized [24] BG particles admixed into adhesive systems have demonstrated the potential to improve the stability of the hybrid layer through mineral deposition and possible inhibition of collagenolytic enzymes.

Designing a BG-based composite material that interacts with dental hard tissues necessitates balancing between two groups of opposed material characteristics, namely mechanical properties and bioactivity. Additionally, the materials should have a sustained bioactive effect, instead of a rapid burst of ions which is soon followed by depletion. To tailor composite properties which would be suitable for a particular purpose, an array of different parameters can be adjusted. The composition of BG can be varied to attain the desired reactivity [12]. Various elements can be added to BG, e.g., silver for an improved antibacterial effect [17], or bismuth for radiopacity [19]. The usual trade-off between bioactivity and mechanical properties can be fine-tuned by varying relative amounts of reinforcing and reactive fillers [25]. Resin matrix composition can be adjusted to optimize hydrophilicity [26] and degree of conversion (DC) [27]. Additionally, BG fillers can be downsized from micrometric to nanometric scale [21,28,29,30,31,32]. Producing nano-sized BG via flame spray synthesis [28] yields primary particles of 20–60 nm [29], unlike the traditional production route by melting and grinding, which gives particle sizes of several micrometers [12]. This downsizing of BG particles enhances their reactive surface area by one to two orders of magnitude [21] and offers new opportunities for designing bioactive materials.

Thus, in the present study, nano- and micro-sized BG particles were admixed into a commercial flowable resin composite. Our aim was to investigate the effect of BG particle size on the following clinically relevant properties: pH elevation, DC, microhardness (MH), and hydroxyapatite precipitation. The null hypothesis assumed that none of the aforementioned properties would be affected by BG particle size.

## 2. Materials and Methods

### 2.1. Preparation of Experimental Composites

Three types of unsilanized glass fillers were used to prepare experimental composites (Table 1). Micro-sized BG 45S5 and inert barium glass produced via melt-quench route were obtained from Schott AG (Mainz, Germany). Nano-sized BG 45S5 was produced by flame spray synthesis as described by Brunner et al. [33]. Briefly, precursors with the corresponding element were mixed and the mixture was fed into a flame reactor. The obtained nanoparticles were collected on a filter above the flame and used as received. Specific surface area and particle size of nano-sized BG were estimated using the Brunauer-Emmett-Teller (BET) method (Micromeritics Tristar, Norcross, GA, USA).

Experimental composites (Table 2) were prepared by admixing 15 wt% of glass fillers into a conventional flowable composite (Heliomolar Flow, Ivoclar Vivadent, Schaan, Liechtenstein; LOT: W28003). According to the manufacturer, Heliomolar Flow contains 40.5 wt% of methacrylate resins (bisphenol-A-glycidyl methacrylate, urethane dimethacrylate, and triethylene glycol dimethacrylate), 59 wt% of reinforcing fillers (silicon dioxide, ytterbiumtrifluoride, and copolymer), and 0.5 wt% of additives (catalysts, stabilizers, and pigments). This commercial composite was chosen as a basis for the preparation of experimental composites because its specific compositional information is provided by the manufacturer. The experimental composites were mixed using a dual asymmetric centrifugal mixer (SpeedMixer DAC 150.1 FVZ, Hauschild Engineering, Hamm, Germany) during one minute at 3500 rpm. The obtained composite pastes were refrigerated in dark containers at 8 °C. At least 24 h before use, the pastes were kept at room temperature (23 ± 2 °C).

For all experiments, disk-shaped specimens (diameter: 6 mm, height: 2 mm) were prepared in custom-made poly(methyl methacrylate) molds, covered by two Mylar strips (Kerr, Orange, CA, USA), pressed between two glass microscope plates, and light-cured for 40 s, as recommended by the manufacturer, using an LED curing unit (Bluephase G2, Ivoclar Vivadent, Schaan, Liechtenstein; 1270 mW/cm^2^) [34]. The radiant exitance of the curing unit was determined using a PM2 thermopile sensor and calibrated FieldMaxII-TO Power Meter (Coherent, Santa Clara, CA, USA).

### 2.2. pH Measurements

Composite specimens (*n* = 3) were immersed immediately after curing in 1 mL of phosphate-buffered saline (PBS, pH: 7.4, 1 specimen per mL, Gibco, Life Technologies, Carlsbad, CA, USA) at 37 °C. The immersion medium remained unchanged throughout the whole measuring period of 28 days. pH changes were consecutively measured using a calibrated pH electrode (780 pH Meter, Metrohm, Herisau, Switzerland) after 0.5, 1, 2, 4, 8 hours, and 1, 2, 4, 7, 14, 21, 28 days. Pure PBS was used as a reference.

### 2.3. Degree of Conversion

The DC at the top and bottom surface of the composite specimens was measured after 24 h of dry storage in the dark at 37 °C and after 28 days of PBS immersion at 37 °C using a Fourier transform infrared (FTIR) spectrometer (Cary 630 FTIR, Agilent Technologies, Santa Clara, CA, USA) with a diamond attenuated total reflectance (ATR) accessory. The FTIR spectrometer was operated under the following conditions: wavelength of 400–4000 cm^−1^, 64 scans, and resolution of 4 cm^−1^. Spectra from uncured composite materials were recorded under the same conditions.

From FTIR spectra collected in absorbance mode, DC was calculated from the changes in the ratio of intensities (peak heights) of aliphatic C=C (1638 cm^−1^) and aromatic C⋯C (1608 cm^−1^) spectral bands using the following equation [35]:

$$\mathrm{DC}\;\left(\%\right)=\left(1\;–\;\frac{{(1638\;\mathrm{cm}}^{-1}{/1608\;\mathrm{cm}}^{-1}){}_{\;\mathrm{after}\;\mathrm{curing}}}{{(1638\;\mathrm{cm}}^{-1}{/1608\;\mathrm{cm}}^{-1}){}_{\;\mathrm{before}\;\mathrm{curing}}}\right)\;\times \;100$$

Six specimens per experimental group were prepared (*n* = 6) and triplicate measurements were performed per surface level of each specimen.

### 2.4. Knoop Microhardness

A digital hardness tester (model no. 1600–6106, Buehler, Lake Bluff, IL, USA) was used to measure Knoop MH on the top and bottom surface of the composite specimens. Measurements were performed after 24 h of dry storage in the dark at 37 °C, and after 28 days of PBS immersion at 37 °C. Indentations were made under a load of 100 g applied for 20 s at random positions around the center of the specimen. The indentations were evaluated with a resolution of 0.015 µm. Six specimens per experimental group were prepared (*n* = 6) and triplicate measurements were performed per surface level of each specimen.

### 2.5. Scanning Electron Microscopy

Composite specimens were placed on aluminum holders using carbon tape and sputtered with 5 nm of tungsten (CCU-010, Safematic, Bad Ragaz, Switzerland) or 10 nm of carbon (MED 020, Bal-Tec, Balzers, Liechtenstein) for scanning electron microscopy (SEM) and energy-dispersive X-ray (EDX) spectroscopy, respectively. The SEM and EDX analyses were performed immediately after specimen preparation and after 28 days of PBS immersion. A scanning electron microscope (Supra 50 VP, Zeiss, Oberkochen, Germany) was operated at 3 kV for monitoring scans to evaluate the surface morphology before and after immersion in PBS. EDX analysis was performed at 10 kV using the X-ray detector X-MAX80 (Oxford Instruments, Abingdon, UK).

### 2.6. Statistical Analysis

Non-parametric statistics were used for the analysis of DC and MH measurements due to violations of parametric assumptions. Kruskal–Wallis rank-sum tests were followed by post-hoc pairwise Wilcoxon rank-sum tests to assess differences between composite materials at the different measuring times, separately for top and bottom data. Resulting *p*-values were adjusted for multiple testing according to Holm. To evaluate intra-group changes in DC and MH values between the measurements at 24 h and 28 days, Wilcoxon signed-rank tests were performed within each material, separately for top and bottom data. Similarly, intra-group comparisons between top and bottom values at a certain measuring time were also assessed using Wilcoxon signed-rank tests. Analyses were performed using the statistical software R (R Foundation for Statistical Computing, Vienna, Austria) [36] including the package tidyverse [37]. The level of significance was set at α = 0.05.

## 3. Results

**Figure 1:**
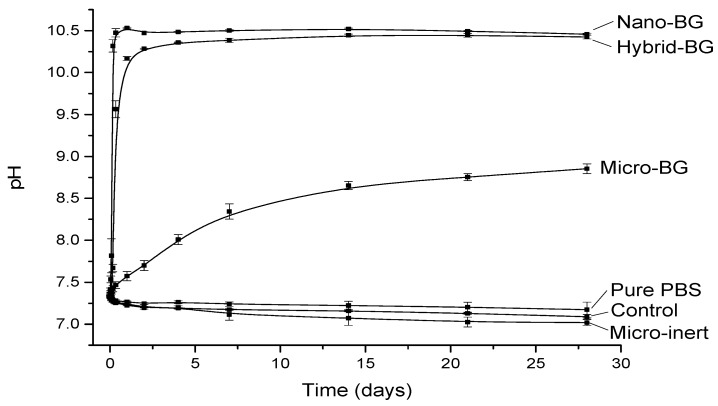
pH (mean ± standard deviation) as a function of immersion time.

The pH changes as a function of time are plotted in Figure 1. All BG-containing composites increased the pH, whereas a slight pH decrease was observed for pure PBS and composites without BG. The fastest pH rise was observed for the nano-BG composite, which increased to 10.5 within the first day of immersion and maintained this pH level throughout the 28-day period. The hybrid-BG composite demonstrated a slightly slower pH rise but eventually reached a similar final value as the nano-BG composite. A considerably slower pH increase and a lower final pH value (8.9) were observed for the micro-BG composite. At the end of the 28-day period, this material still showed a slight tendency of pH increase.

**Figure 2:**
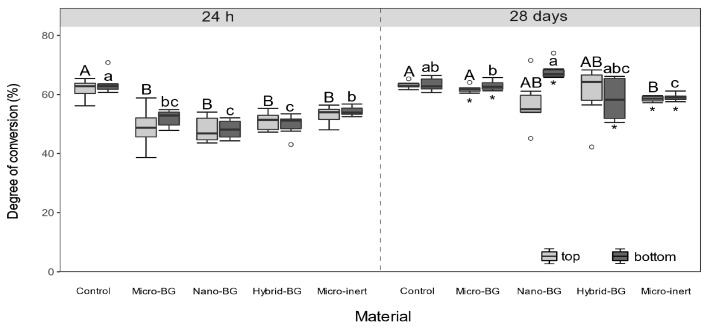
Degree of conversion measured after 24 h and 28 days. Within each measuring time, same uppercase letters indicate statistically homogeneous groups for top surfaces, while same lowercase letters indicate statistically homogeneous groups for bottom surfaces. Asterisks (*) indicate significant differences between measuring times, within each material/surface combination.

**Figure 3:**
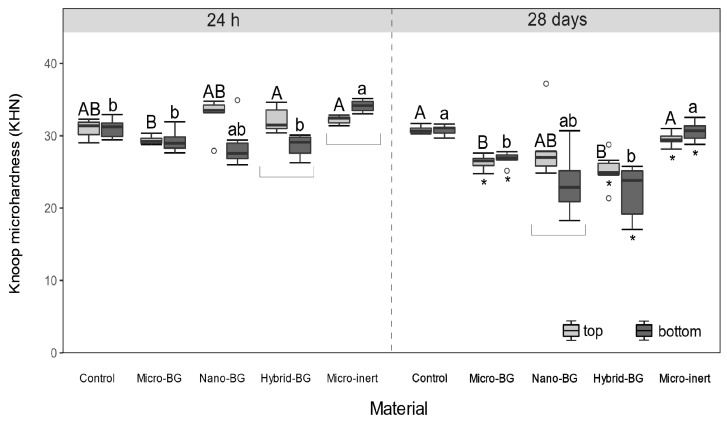
Knoop microhardness measured after 24 h and 28 days. Within each measuring time, same uppercase letters indicate statistically homogeneous groups for top surfaces, while same lowercase letters indicate statistically homogeneous groups for bottom surfaces. Asterisks (*) indicate significant differences between measuring times, within each material/surface combination. Square brackets indicate significant differences between the top and bottom surfaces.

Experimental composites showed significantly lower initial (24 h) DC values than the control composite at both top (*p* = 0.02–0.04) and bottom (*p* = 0.02) surfaces (Figure 2). After 28 days, DC differences between the BG-containing composites and the control composite were leveled (*p* > 0.05). Figure 3 indicates that initial MH values of BG-containing composites were similar to that of the control composite (*p* > 0.05), despite the aforementioned DC decline caused by the additional fillers. After 28 days of PBS storage, MH values of the micro-BG, hybrid-BG, and micro-inert composites were diminished (*p* = 0.03). Variations in the BG particle size among the micro-BG, nano-BG, and hybrid-BG composites had generally little effect on DC and MH.

**Figure 4:**
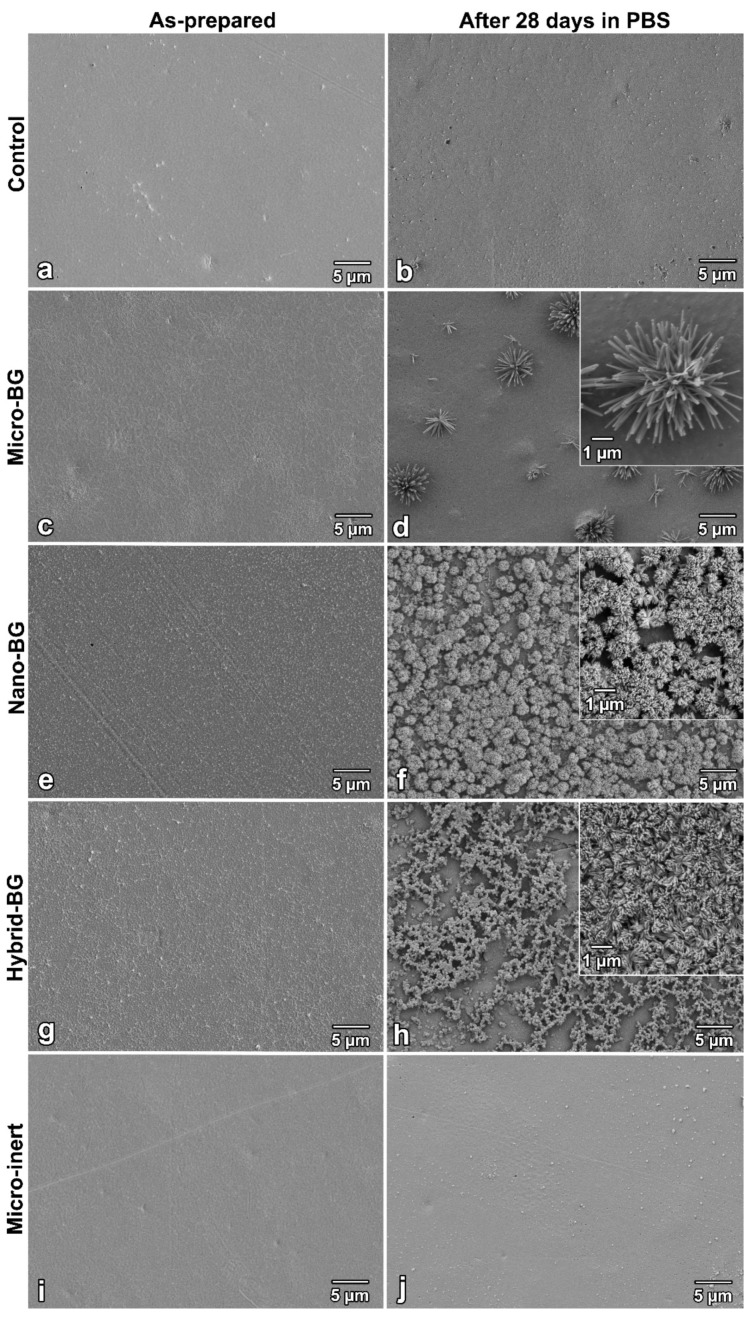
Scanning electron microscopy images of specimen surfaces immediately after preparation and after 28 days of storage at 37 °C in phosphate-buffered saline (PBS) for the control (**a**,**b**), micro-BG (**c**,**d**), nano-BG (**e**,**f**), hybrid-BG (**g**,**h**), and micro-inert composites (**i**,**j**). Insets show higher magnification of crystals formed on specimen surfaces.

SEM images of composite specimens before and after 28-day PBS immersion are shown in Figure 4. The control and micro-inert composites showed smooth surfaces before and after PBS immersion. Evenly distributed nano-sized BG particles can be seen in as-prepared specimens of the nano-BG and hybrid-BG composites. After 28 days of PBS immersion, a crystalline precipitate was identified on the surfaces of all BG-containing composites. Crystals formed by micro-sized BG were visibly larger and less dense than those formed by nano-sized BG.

**Figure 5:**
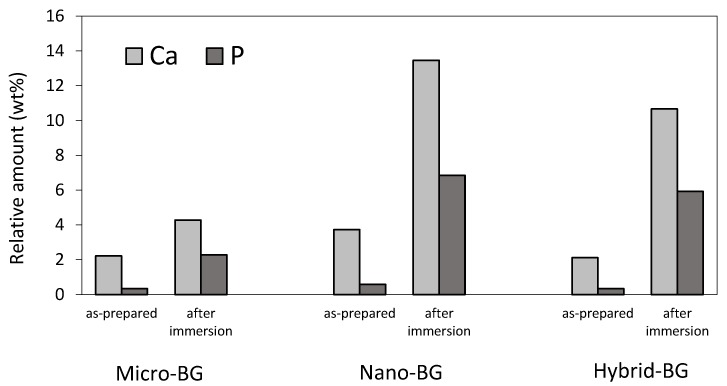
Relative amounts (in weight %) of calcium and phosphorus identified on the surface of BG-containing composites before and after PBS immersion.

Figure 5 shows the EDX data collected from the surface of BG-containing composites. An increase in calcium and phosphorus concentrations indicative of calcium phosphate precipitation was observed after 28 days of PBS immersion. The increase in calcium and phosphorus concentrations was more pronounced for the nano-BG and hybrid-BG composites compared to the micro-BG composite. The Ca/P ratio in the range of 1.39–1.52 suggests that the precipitate was calcium-deficient hydroxyapatite. On the surfaces of the control and micro-inert composites, no measurable amounts of calcium and phosphorus were detected both before and after PBS immersion.

## 4. Discussion

This study investigated the effect of admixing micro- and nano-sized BG into a commercial flowable resin composite. Indicators of bioactivity (pH increase and hydroxyapatite precipitation) and basic physico-chemical properties (DC and MH) were investigated. The null hypothesis was partially rejected because nano-sized BG fillers accelerated the pH increase and improved hydroxyapatite precipitation, but generally had no effect on DC and MH. While some experimental designs use exclusively experimental composites with varying BG amounts [13,15], the approach of doping a commercial composite with BG is beneficial because baseline values of evaluated properties correspond to the material which has been established clinically.

Various benefits of the high surface area of nano-sized BG particles have been reported. A study comparing the effect of nano- and micro-sized BG for endodontic purposes showed that the enhanced surface area of nano-sized particles accelerated BG dissolution resulting in higher ion release and pH elevation, and, ultimately leading to a better antimicrobial effect [29]. Similarly, a study comparing micro- and nano-sized particles of bismuth-containing BG incorporated in a commercial epoxy resin root canal sealer showed faster pH elevation and hydroxyapatite precipitation for nano-sized particles [19]. Nano-sized BG has also been shown to induce a higher dentin remineralization rate than micro-sized BG [32]. Benefits of the high surface area of nano-sized BG have also been investigated beyond the field of dental materials, for example in designing biodegradable composites for tissue engineering [30,31]. Other types of nano-sized reactive fillers, e.g., hydroxyapatite, have also been incorporated into restorative dental materials [8].

The nano-BG composite induced a considerably faster pH increase than the micro-BG composite. This finding can be explained by the fact that nano-sized BG particles have an about 30 times larger specific surface area available for ion exchange. The higher reactivity of nano-sized vs. micro-sized particles has also been reported in a study on antimicrobial properties of BG 45S5 [29]. Additionally, studies on experimental composites functionalized with amorphous calcium phosphate have shown that downsizing of reactive fillers can improve the mechanical properties of resin composites, while maintaining or improving their ion release [38,39]. It is interesting to note that the pH increase caused by the hybrid-BG composite had similar kinetics and extent as that of the nano-BG composite. This suggests that at least half of the total amount of nano-sized BG can be replaced by micro-sized BG with practically no negative effect on the composite’s capability to elevate pH. Such a fine-tuning of the relative amounts of nano- and micro-sized BG should help in controlling the effect of nanoparticles on material viscosity [21] and finding a balance between bioactivity and mechanical properties.

The absolute values of pH increase are difficult to compare among different studies due to varying types and volumes of immersion media, as well as different ratios of composite specimen surface area to immersion media volume [12,40]. However, the evaluation of pH changes in a closed system can, to a certain extent, simulate conditions within the marginal gap. The small volume within the marginal gap produces conditions which may be even more conducive for the pH increase than those used in the present study (specimen surface area of 94.2 mm^2^/1 mL of PBS). Although the immersion medium was not replenished during the 28-day study period, its volume per surface area was much higher than the volume of saliva which can be expected to be present in the marginal gap. Despite an obvious simplification, this higher volume can simulate the inflow of a fresh solution into the marginal gap.

The comparison of 24-h DC values between the control and experimental composites shows that DC was diminished to a similar extent by the addition of 15 wt% of fillers, regardless of filler type. This indicates that the resin mobility was not hindered by the high surface area of nano-sized BG any more than the 30 times lower surface area of micro-sized particles. A dose-dependent potential of BG 45S5 for diminishing the DC of experimental composites has been observed in a previous study [27]. The present study demonstrated that downsizing the BG fillers exerted no inhibitory effect on resin polymerization. Most of the initial DC differences between the control and experimental composites were leveled after 28 days due to post-cure polymerization [41]. At that time, no differences were identified between the control composite and BG-containing composites. It should be mentioned that the DC values of all composites were within the range reported for commercial composites [42], regardless of material composition and layer thickness. Additionally, the top and bottom DC values were statistically similar for all pairwise comparisons, indicating that a homogeneous polymerization was attained throughout the 2 mm thick composite layer.

The decline in 24-h DC caused by additional fillers did not translate into diminished 24-h MH values. It appears that the DC decline in experimental composites was counterbalanced by the increased filler loading [43], resulting in unchanged MH values. Mechanical properties of resin composites are generally improved by higher filler loadings [44] and can be additionally improved by coating filler particles with reactive silanes [45]. However, reactive particles such as BG are usually used unsilanized [12,13,15,25] because hydrophobic silane coating can impair their reactivity and diminish bioactive properties [46]. This was the rationale for using unsilanized fillers in the present study. The addition of unsilanized BG fillers to resin composites has been demonstrated to diminish mechanical properties in a dose-dependent manner and contribute to more extensive material degradation in an aqueous environment [25]. Although in the present study such a negative effect was not identified by MH measurements after 24 h, the results obtained after 28 days of PBS immersion indicated a decrease in mechanical properties of BG-containing composites. A decrease in mechanical properties due to an aqueous immersion has been commonly observed for composites functionalized with soluble fillers [47], as well as for conventional composites containing only reinforcing fillers [48].

After 28 days of PBS immersion, the micro-inert composite showed similar MH as the control composite, whereas the MH of the micro-BG and hybrid-BG composites was lower than that of the control. This finding was expected because the degradation of BG fillers in an aqueous medium is known to diminish mechanical properties [25,49]. However, 28-day MH values were statistically similar among all BG-containing composites, indicating that downsizing the BG particles neither improved nor diminished the composite’s MH. Pairwise comparisons of MH values before and after BG immersion identified a significant MH decrease for all experimental composites, except for the nano-BG composite. Although the nano-BG composite showed MH values in the range of the other BG-containing composites, the statistical analysis did not reveal a significant effect of PBS immersion due to high data variability for this particular material. The comparatively higher scattering of MH data observed for the nano-BG composite may be attributed to uncontrolled agglomeration of nanoparticles and consequently higher heterogeneity of local mechanical properties [50].

The comparison of MH between the top and bottom surfaces identified lower bottom values for hybrid-BG after 24 h and nano-BG after 28 days. These findings show that MH measurements were more capable of detecting curing heterogeneity through a 2 mm thick layer than DC measurements [51], which revealed no differences. The micro-inert composite showed significantly higher initial bottom MH, which is a phenomenon occasionally found in similar study designs [34] and is usually explained by the intrinsic heating within the composite specimen [52].

A crystalline layer resembling hydroxyapatite was observed on surfaces of all BG-containing composite specimens after 28 days of PBS immersion. The nano-BG and hybrid-BG composites formed smaller and more evenly distributed crystals compared to the composite filled with micro-BG. This produced a more homogeneous coverage of the nano-BG and hybrid-BG composite surfaces, which may be beneficial for marginal gap sealing [16]. The EDX analysis suggested the presence of a hydroxyapatite layer by detecting an increase in calcium and phosphate concentration after 28 days of immersion. A more pronounced increase in calcium and phosphorus concentrations on the surface of composites containing nano-sized BG fillers is in agreement with a more densely covered surface observed by SEM.

The hybrid-BG and nano-BG composites showed similar results with regards to all measured properties: pH increase, hydroxyapatite precipitation, DC, and MH. This finding indicates that nano-sized BG can be partly replaced by micro-sized BG, without negatively affecting any of the investigated properties. The potential to adjust relative amounts of differently sized particles is important because nano-sized BG can limit material usefulness by diminishing the amount of fillers that can be incorporated into the composite [50]. Another potential problem associated with using exclusively nano-sized BG is its high initial reactivity accompanied by a failure to maintain the bioactive effect over time [21]. Therefore, one can envision possible benefits of a hybrid material in which nano-sized BG would ensure fast precipitation of the hydroxyapatite layer for immediate sealing of the marginal gap, whereas less reactive micro-sized BG would ensure a long-term ion releasing and alkalizing effect [21,29].

Because BG 45S5 is highly reactive, more stable low-sodium BG compositions have recently been suggested as potential alternatives for use in resin composites [12,15]. BG compositions of lower reactivity can ensure a more sustained remineralizing effect and help in maintaining mechanical properties over time [15]. Additionally, BG compositions can be modified to release therapeutic ions, such as fluorides [12]. Since the present study documented a favorable effect of nano-sized BG 45S5, potential benefits of nanoparticles of various novel BG compositions produced by flame spray or sol-gel route should be investigated in future work. As BG has shown excellent biocompatibility in orthopedic applications [53], it is unlikely to exert possible toxic effects on oral tissues when incorporated into resin composites [54].

Overall, this study indicated that downsizing the BG fillers to nano-size can be used to improve the reactivity of experimental composites without sacrificing their fundamental properties. However, further investigations on pH induction, mechanical properties and bioactive effects under clinically relevant conditions are needed. In this study, an indication of hydroxyapatite precipitation was observed under static conditions in which a simplified immersion medium was used without being replenished for 28 days. The clinical situation dictates a more complex environment with varying ionic strengths, continuous flow of immersion solution, and periodic pH drops produced by acidogenic bacteria. All of these factors can affect the reactions occurring on the material surface, as well as the potential remineralization of dental hard tissues. Additional in-depth studies are required to confirm and further characterize the capability of the BG-functionalized composites for hydroxyapatite precipitation.

## 5. Conclusions

Within the limitations of this in vitro study, it is concluded that downsizing bioactive glass particles to nano-size improves the alkalizing and hydroxyapatite-forming potential of experimental composites with no negative effects on their degree of conversion and microhardness. The demonstrated benefits of using nano- over micro-sized bioactive glass as a reactive filler for dental composites will be useful to fine-tune filler particle size in future studies. Additionally, clinical studies should be performed to evaluate the caries-protective effects of resin composites functionalized with bioactive glass.

## Figures and Tables

**Table 1:** Bioactive and inert glass fillers.

	Micro-Sized Bioactive Glass 45S5	Nano-Sized Bioactive Glass 45S5	Micro-Sized Inert Barium Glass
Mean Particle Size (µm)	5.6	0.07	6.7
Specific Surface Area (m^2^/g)	1.1	35.3	1.7
Composition (wt%)	45% SiO_2_	45% SiO_2_	55% SiO_2_
	24.5% CaO	24.5% CaO	25% BaO
	24.5% Na_2_O	24.5% Na_2_O	10% Al_2_O_3_
	6% P_2_O_5_	6% P_2_O_5_	10% B_2_O_3_
Product Name/LOT	G018-144/SM528	experimental batch	GM27884/M92605

**Table 2:** Composition of experimental composites.

Material Designation	Composition (wt%)
Control	100% Heliomolar Flow
Micro-BG	85% Heliomolar Flow + 15% micro-sized BG
Nano-BG	85% Heliomolar Flow + 15% nano-sized BG
Hybrid-BG	85% Heliomolar Flow + 7.5% micro-sized BG + 7.5% nano-sized BG
Micro-inert	85% Heliomolar Flow + 15% micro-sized inert barium glass

BG: bioactive glass 45S5.
